# Sublingual immunotherapy for pediatric patients with mite allergies

**DOI:** 10.1097/MD.0000000000028690

**Published:** 2022-01-28

**Authors:** Teruyuki Kajiume

**Affiliations:** Mukainada Child Clinic, 24–26 Aosaki-naka, Fuchu-cho, Aki-gun, Hiroshima, Japan.

**Keywords:** allergy and immunology, child, drug-related side effects and adverse reactions, house dust mites, sublingual immunotherapy

## Abstract

Sublingual immunotherapy (SLIT) has been increasingly used instead of subcutaneous immunotherapy. SLIT was initially approved for use among adults; however, it has become more widely accepted for children. Few studies have evaluated the effectiveness of SLIT in the treatment of dust mite allergies among children, including adverse effects. This study aimed to investigate the effectiveness of SLIT in children with dust mite allergies, as well as its adverse effects, at a pediatric general outpatient clinic.

I analyzed the data of 181 patients aged 4 to 12 years who tested positive for mite antigen-specific immunoglobulin E, exhibited nasal and/or eye symptoms, and received Miticure. Symptoms were evaluated using the Japanese rhino-conjunctivitis quality of life (QOL) questionnaire no. 1. Wilcoxon tests were used to compare the pretreatment and post-treatment symptom scores. Adverse events were tallied, and Kaplan–Meier curves and Wilcoxon tests were used to assess the proportion of dropouts.

The mean QOL score at the baseline was 2.17 (standard deviation [SD] 1.45). After 1 week, the mean symptom QOL score was 1.63 (SD 1.32); the lowest mean score was found in week 41 (0.48, SD 0.63). A significant decline in the occurrence of all symptoms, including sneezing, nasal discharge, nasal congestion, itchy eyes, and teary eyes, was observed. Adverse effects were observed in 76 (42.0%) patients; the most common adverse effect was itchy mouth.

SLIT improves symptoms with minimal adverse effects in pediatric patients.

## 1 Introduction

Allergen immunotherapy (AIT) is a radical treatment modality that improves the natural progression of allergies in patients.^[1]^ In recent years, many regions, particularly Europe, have adopted sublingual immunotherapy (SLIT) instead of the conventional subcutaneous immunotherapy (SCIT) for the treatment of allergic rhinitis and asthma.^[2,3]^ SLIT has been rapidly accepted primarily due to the reduction in adverse events and the convenience of administration. SLIT was initially approved for adults; however, it has become more widely accepted for children.

In 2014 and 2015, Japan approved the use of cedar pollen sublingual extract and sublingual tablet, respectively, for the treatment of house dust mite allergies. In February 2018, pediatric indications were also approved, which removed the restriction limiting the treatment to patients aged ≥12 years. SLIT is clearly less invasive than the conventional hyposensitization therapy in the form of SCIT, and the insurance coverage for children encourages its use.

Recent reviews have concluded that SLIT effectively reduces the occurrence of symptoms, particularly those of allergic rhinitis, in pediatric populations. However, they have also suggested that further trials comparing the efficacy of SCIT with that of SLIT are needed^[4]^ and that the current results are more convincing for pollen allergy than for dust mite allergy.^[2,5]^ Some studies comparing the results of SLIT between adults and children suggest that SLIT is equally effective in both the groups. A Korean study comparing the results of SLIT for the treatment of dust mite-sensitized allergic rhinitis in children (n = 54) and adults (n = 22) found similar levels of improvement in both groups and no major differences in improvement outcomes between school-age and adolescent children. However, SLIT led to better improvements in children than in adults.^[6]^ A recent trial in Brazilian children indicated that SLIT was effective in reducing atopic dermatitis in patients sensitized to dust mites.^[7]^ An international randomized phase III trial concluded that SLIT was effective in reducing allergic rhinitis symptoms and medication use, and improving quality of life (QOL) compared to placebo in adolescents and adults with sensitization to dust mites.^[8]^ There is evidence than SLIT for dust mite sensitization may prevent progression from allergic rhinitis to allergic asthma.^[9]^ Dust mite SLIT has been shown to inhibit epitope spreading and increase blocking antibodies in preschool children.^[10]^ However, use of SLIT is controversial for allergic asthma; studies have not always distinguished between single-allergen and multiallergen interventions, nor tablet and droplet preparations,^[11]^ and SCIT may be more effective in reducing medication use.^[12]^

Adverse drug reactions have been a concern of SLIT treatment. In 1 study of Japanese children, 46.5% experienced an adverse reaction during the first 4 weeks of therapy; most reactions were mild, including itching of the oral mucosa and ears, and throat irritation.^[13]^ Among UK children, SCIT and SLIT both improved QOL, but children receiving SLIT were more likely to discontinue treatment, often due to flares of atopic dermatitis, while asthma flares were more likely with SCIT.^[14]^

Few studies have evaluated the effectiveness of SLIT in the treatment of dust mite allergies among children, including adverse effects. Thus, this study presents the results of SLIT recorded at a general practice pediatric clinic.

## 2 Methods

### 2.1 Patients

The present study was conducted at a single primary care clinic in Hiroshima, Japan. Patients were eligible for inclusion if they tested positive for class ≥2 mite antigen-specific immunoglobulin E (IgE). Antigen-specific IgE was identified via chemiluminescent enzyme immunoassay and nonspecific IgE was determined via fluorescence enzyme immunoassay^[15]^ (Fukushima Rinsho, Hiroshima, Japan). This study also included patients who exhibited nasal and/or eye symptoms, had received house dust mite sublingual tablets (Miticure; Torii Pharmaceutical, Tokyo, Japan) between February 16, 2018 and March 31, 2021, and were younger than 13 years of age at SLIT initiation. This report included all patients who visited the clinic and met the inclusion criteria. SLIT was initiated upon request and consent from the guardian. In accordance with the dosing strategy for Miticure, a 10,000 Japanese allergy unit tablet was continuously administered after administering a 3300 Japanese allergy unit tablet for 1 week. There were no restrictions on other treatments for allergic rhinitis, including those administered at other hospitals, and treatments were not excluded on the basis of comorbidities. No detailed information on medications prescribed at other clinics was available, although antihistamines and leukotriene receptor antagonists were the most commonly prescribed medications. Other conditions, such as severe bronchial asthma or atopic dermatitis, could have been observed at a specialty clinic but were not necessarily recorded in the medical records at our clinic. Patients who refused SLIT for any reason were not included in this analysis, and those who stopped attending after the first 7 days were not followed up for results or adverse effects. The median patient age was 7 years 11 months (range: 4 years 0 months to 12 years 11 months); 102 patients were boys and 79 patients were girls.

### 2.2 Evaluation

Patients were followed up for routine clinical care, with return visits every 4 to 8 weeks. Data on dropouts were obtained until the patient dropped out. Symptoms were evaluated using the Japanese rhino-conjunctivitis QOL questionnaire no. 1, a standard and validated measure that is widely used in Japan.^[16,17]^ This tool includes a series of questions regarding the severity of multiple symptoms and the extent of its effects on several domains of QOL. It is preceded with a description that explains that the purpose of the survey is to determine to what extent the rhinitis interferes with their life and whether it would be improved by treatment; participants are told that they may find some of the questions difficult to answer, but they should just answer to the best of their ability. Participants were asked to rate the severity of the worst nasal and eye symptoms they had experienced over the past 1 to 2 weeks. Five items (sneezing, nasal discharge, nasal congestion, itchy eyes, and teary eyes) were evaluated on a five-point scale (0 = no symptoms, 1 = mild, 2 = somewhat severe, 3 = severe, and 4 = extremely severe) at each consultation before treatment initiation, and the symptom scores were calculated and summed to obtain a total score. Parents reported symptoms if the patient was too young. Otherwise, patients reported their own symptoms if they were able to respond. The choice of reporting of symptoms by the patient or parent was based on the patient's situation rather than a specific age cutoff.

### 2.3 Statistical analysis

Paired *t* tests were used to compare the pretreatment and post-treatment symptom levels (all comparisons were conducted on the same participants, rather than comparing across groups). Adverse events were tallied, and Kaplan–Meier curves and *t* tests were used to compare dropout rates between patients with and without adverse effects and between patients with and without symptom relief. The results were also stratified by age (4–6, 7–8, 9–10, and 11–12 years) and compared using the *t* test or Fisher exact test, as appropriate.

Information regarding the study was presented to the parents of the patients at the hospital; the opt-out method was used to guarantee the study participants the opportunity to refuse. The study protocol was approved by the Institutional Review Board of the Hiroshima Prefecture Medical Association (approval no. 004).

The statistical software BellCurve for Excel (Social Survey Research Information Co., Ltd, Tokyo, Japan) was used. When two-tailed *P*-value was <.05, it was considered statistically significant.

## 3 Results

**Figure 1:**
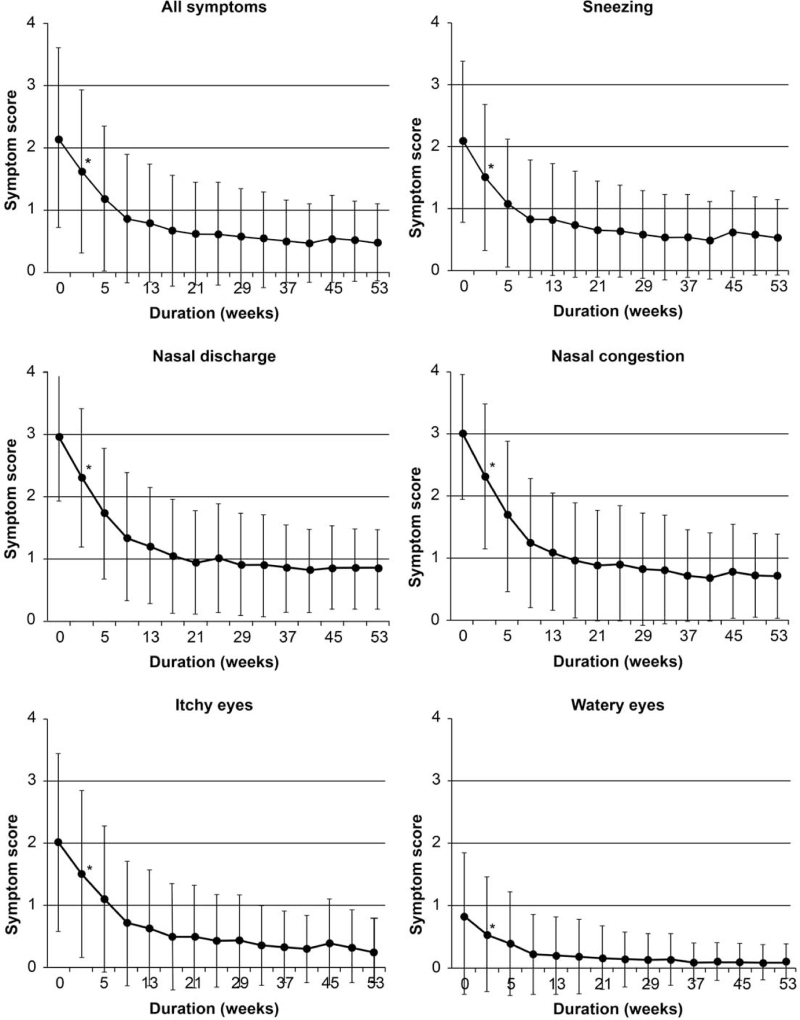
Mean symptom scores among patients who underwent sublingual immunotherapy. Statistically significant (*P* < .05) findings are starred at only place of 1 week, though there were significant difference after 5 weeks as same as 1 week. The average score of 5 symptoms, and the scores of each of the 5 symptoms, all improved at 1 week from start of SLIT.

The mean QOL score at the baseline was 2.17 (standard deviation [SD] 1.45). After 1 week, the mean symptom QOL score was 1.63 (SD 1.32); the lowest mean score was found in week 41 (0.48, SD 0.63). A substantial decline was observed for all symptoms, including sneezing, nasal discharge, nasal congestion, itchy eyes, and teary eyes (Fig. 1). Nasal discharge and nasal congestion symptom scores were higher than those of other symptoms at the baseline, while those for eye symptoms were lower at baseline; however, a significant effect was observed for all symptoms. No differences were observed between age groups (*P* > .05).

Adverse effects were observed in 76 of 181 (42.0%) patients. The most common adverse effect was itchy mouth (n = 56, 30.9%), followed by stomatitis (n = 20, 11.0%), itchy ears (n = 18, 9.9%), itchy body (n = 2, 1.1%), itchy eyes (n = 3, 1.7%), and nausea (n = 1, 0.6%) (Table 1). All adverse effects were local rather than systemic and resolved (generally within a few months) as SLIT continued. No differences were observed between age groups (*P* > .05).

**Table 1:** Adverse effects reported among 181 pediatric patients receiving sublingual immunotherapy.

Symptoms	Number of patients	Total number of patients	Adverse effect rate
Overall	76	181	42.0%
Itchy mouth	56	181	30.9%
Stomatitis	20	181	11.0%
Itchy ears	18	181	9.9%
Itchy body	2	181	1.1%
Itchy eyes	3	181	1.7%
Nausea	1	181	0.6%

**Figure 2:**
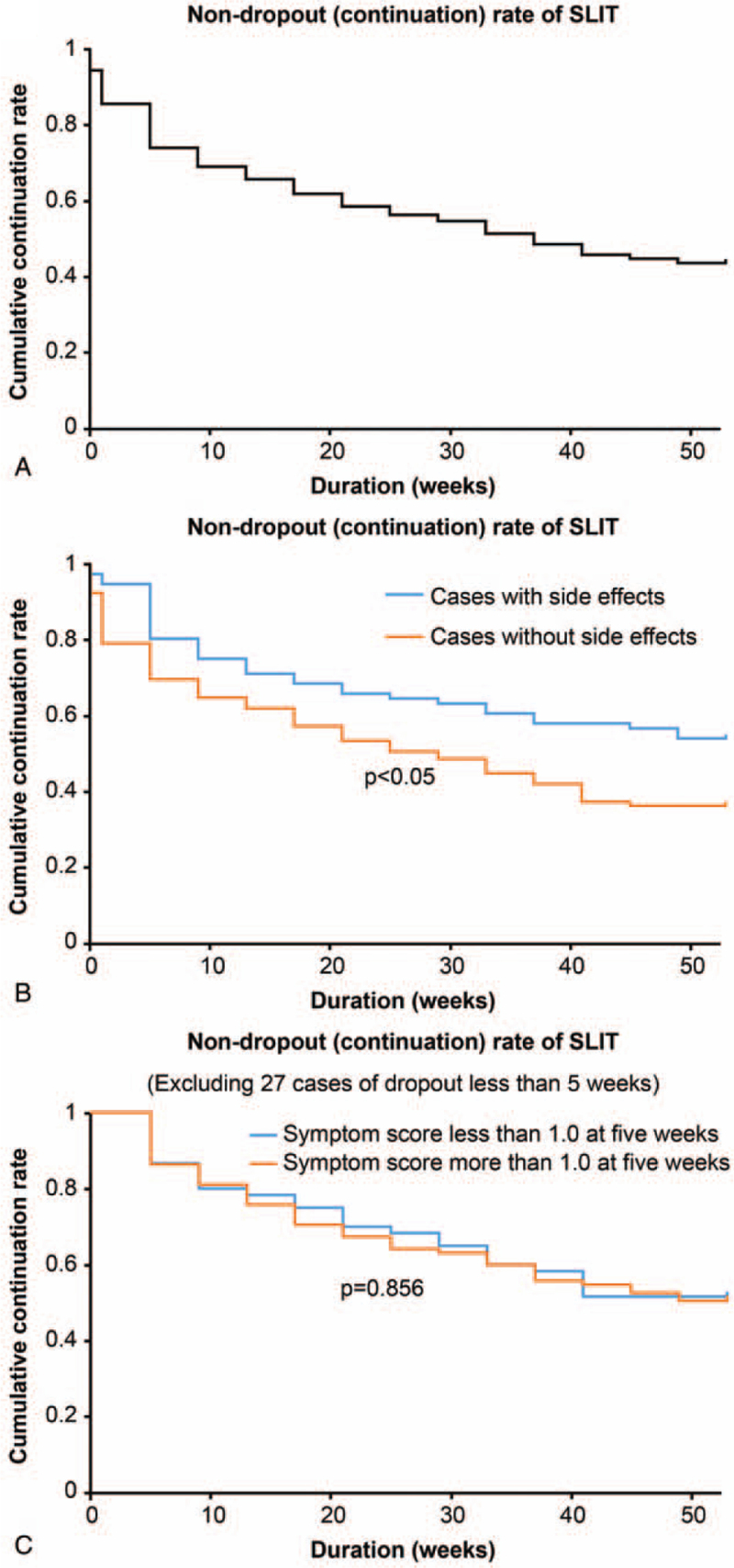
Continuation of sublingual immunotherapy (SLIT) by adverse effects and symptom score. A: It was shown the overall dropout rate. 43.6% of patients have been on for more than a year Most of dropped-out cases in about one month from start of SLIT, and then gradually decreased. 43.6% of patients have been on for more than a year. B: It was shown the dropout rate separately with and without side effects. The rate of dropouts was greater among patients without adverse effects than among those with adverse effects (*P* < .05). C: It was shown the dropout rate separately more than 1 or less of symptom score at 5 weeks. There was no significant difference.

**Figure 3:**
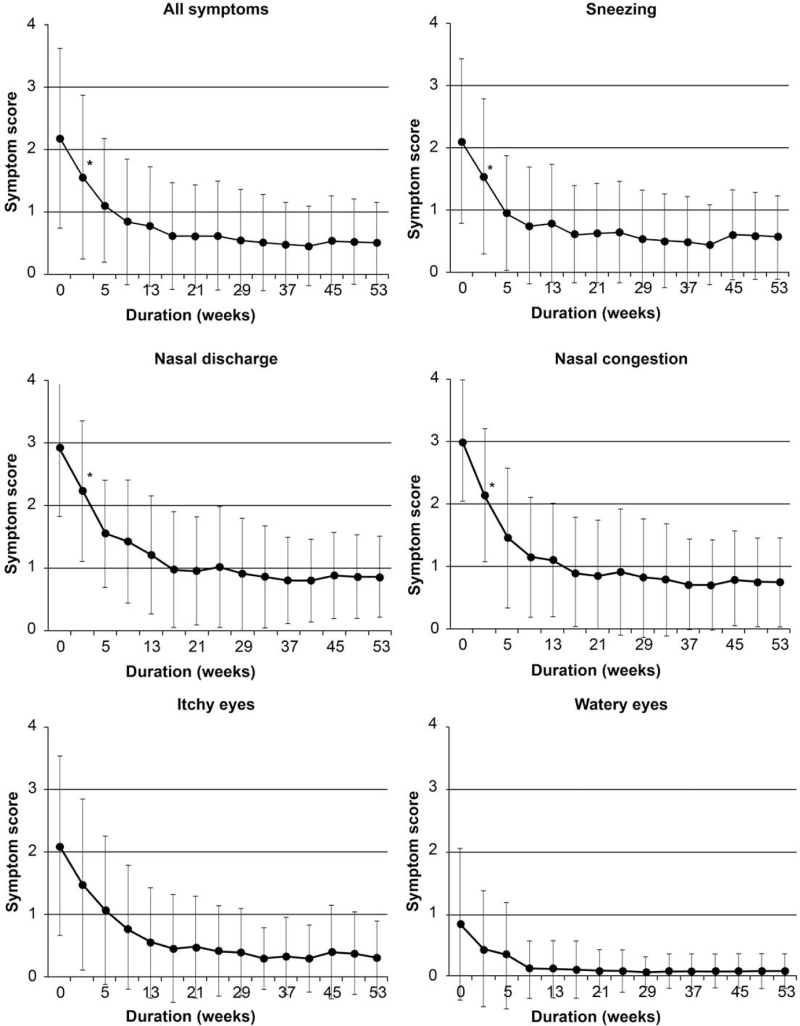
Symptom scores among patients who continued treatment for >1 year. Statistically significant (*P* < .05) findings are starred at only place of 1 week, though there were significant difference after 5 weeks as same as 1 week. The average score of 5 symptoms, and the scores of each of the 5 symptoms, all improved at 1 week from start of SLIT.

Seventy-nine (43.6%) patients continued therapy for >1 year (Fig. 2A). Surprisingly, the number of dropouts was greater among patients without adverse effects than among those with adverse effects (*P* = .012) (Fig. 2B). In addition, there was no difference in dropout rates between patients with higher symptom scores after the baseline and those with lower symptom scores after the baseline (*P* = .789) (Fig. 2C). Among the patients treated for >1 year, there was a significant decline in the overall score from 2.20 (SD 1.41) to 1.55 (SD 1.30) in week 1. This trend extended to all symptoms, including sneezing, nasal discharge, nasal congestion, itchy eyes, and teary eyes (Fig. 3).

## 4 Discussion

House dust mite allergies are highly prevalent and major contributors to sensitization and atopy. AIT has been used for approximately 100 years, and its efficacy against allergic rhinitis and bronchial asthma has been widely appreciated.^[1,18]^ SCIT has been used since the 1960s; however, the number of SCIT-treated cases has been declining due to the possibility of occasional serious allergic reactions. In the 1980s, in an attempt to administer AIT safely, SLIT was developed,^[10]^ and thereafter, it gradually replaced SCIT.^[3]^ House dust mite allergy is a condition that has a great social impact; the sensitization rate is very high, and there are many reports on its relationship with the acquisition of atopic constitution in childhood.^[19]^ Miticure is a SLIT formulation containing a house dust mite allergen developed by ALK-Abelló A/S in Denmark and is a standard drug worldwide. In February 2018, Japan was the first country worldwide to approve the administration of this drug to children aged <12 years. This study showed that symptoms in pediatric patients declined significantly and rapidly with the use of SLIT. Symptom scores improved much earlier than expected; significant effects were observed from the first week, especially for nasal symptoms, and the effect on nasal congestion was particularly remarkable. This is notable, as several previous studies have suggested that improvement with SLIT may be delayed, especially compared to SCIT.^[20]^ When the effect of AIT is evaluated based on the skin reaction, the effect in the late phase appears earlier than that in the immediate phase.^[21]^ Sneezing and nasal discharge appear 1 to 2 minutes after antigen induction, whereas nasal congestion appears slightly later and lasts for 5 to 6 hours; therefore, it is considered a late phase response. The positive effect on nasal obstruction observed in this study may be the result of the effect of AIT in the late phase. Eye symptom scores improved, but not markedly. One study indicated that SLIT induced changes in dendritic cell function, possibly related to T cell activation and regulatory function.^[22]^ SLIT may promote the formation of Th1 cells, inhibit Th2 cells, and restore the Th1/Th2 balance by regulating cytokine expression.^[23]^ SLIT also appears to induce regulatory T cell suppression via interleukin-10^[24]^ thus assisting in the control of potentially harmful T cells.

Previous studies have generally supported the efficacy of SLIT in pediatric allergies. A review conducted in 2009 reported strong evidence regarding its efficacy in pollen allergy, but conflicting evidence for dust mite allergies.^[25]^ A randomized, placebo-controlled trial conducted among Japanese children aged 5 to 17 years found that daily administration of house dust mite tablets was associated with a reduced total combined rhinitis score (symptoms and medications) and was effective in both children and adolescents.^[26]^ A randomized, placebo-controlled trial of 15 Turkish children found that SLIT improved the symptoms of rhinitis and asthma.^[27]^ A trial comparing SLIT to SCIT and pharmacotherapy found that both SLIT and SCIT were associated with clinical improvement in rhinitis and asthma; however, severe adverse events were more common with SCIT.^[20]^ A Japanese placebo-controlled trial of SLIT in 31 subjects aged 7 to 15 years with dust mite allergic rhinitis found that symptoms were reduced after 24 weeks of treatment and that significant differences were observed after 30 weeks of treatment.^[28]^ Another 2-year trial involving 282 pediatric allergic rhinitis patients that compared SCIT and SLIT to a placebo found a reduction in the clinical symptom score; it was more pronounced with SLIT after 2 years of treatment and with SCIT after the first year.^[29]^ One year after treatment with SLIT, symptoms receded significantly compared to those before treatment. However, there was no significant difference between symptoms observed 1 and 2 years after treatment.^[30]^ A study of SLIT among 39 pediatric patients allergic to dust mites with a mean age of 8.8 years examined results after 3 years of therapy and found remission of rhinitis in 82% of patients.^[31]^ In a retrospective study of 102 cases, symptoms significantly receded post-SLIT, and the peripheral blood eosinophil counts and serum IgE levels also significantly decreased.^[23]^

The adverse effects of SLIT have been reported to be mild. A recent randomized controlled trial including 438 patients found that almost all patients experienced adverse events, accounting for 96.8% of patients in the SLIT group and 94.5% patients in the placebo group over the course of a year.^[32]^ This might have been because it included infections that had no causal relationship with SLIT. Treatment-related adverse events accounted for 67.1% of the reported adverse events; the most common adverse events were mouth edema, throat irritation, ear pruritus, and mouth swelling. The reported events were similar in this study, but were less common. This may be due to the detailed level of observation in specialized outpatient clinics, unlike that in general clinics. Other trials have reported no adverse effects.^[27]^ Sublingually administered allergens are not absorbed into the circulation,^[33]^ and absorption into the body is almost limited to phagocytosis of dendritic cells under the tongue, which may be the rationale for the few severe adverse effects.

The dropout rate was relatively high in the present study. This may be due to the burden of administration – SLIT tablets must be kept under the tongue for 1 minute before swallowing. In addition, as this study was conducted at a general outpatient clinic and not an allergy clinic, there may have been a higher rate of patients with only mild mite allergies. In addition, many patients do not complete the course of therapy once the symptoms have remitted and prefer not to follow a 3-year course of administration. However, dropout was not associated with intolerable adverse effects or poor results. Rather, many adverse effects were observed in the continued SLIT group. The patients may have dropped out before experiencing any adverse effects. Furthermore, few patients dropped out due to intolerable adverse effects. The rapid improvement in the symptoms may be partly due to other medications received for allergic rhinitis, and as these were prescribed at another clinic, their effects could not be assessed or controlled. In general practice, patients are advised to discontinue antihistamine treatment once symptoms have improved, and most patients ultimately undergo SLIT only. The effects of treatment normally appear in a week, and significant effects can be expected with continued use for at least 1 year. The manufacturer recommends continuing the medication for 3 years; however, in the future, it will be necessary to consider the recurrence rate among dropouts.

The strengths of this study include the setting of a general practice nonspecialty clinic, indicating real-world results of treatment, and the assessment of patient-relevant outcomes with a validated instrument. The limitations include the lack of a placebo or other control group, the high dropout rate, limited additional data on participants (such as other treatments), and the fact that no data obtained at a single clinic can be truly generalizable. Severe bronchial asthma is a contraindication for use according to the manufacturer; however, information on this and other comorbidities was not collected in this study.

The present report reveals the real-world situation of SLIT administration among children younger than 12 years in actual clinical practice in general pediatric clinics. This study provides useful information for pediatricians and other practitioners who are considering SLIT administration among children aged <12 years. Future research should include studies that determine which patients benefit most from treatment, whether patients with comorbidities can benefit (for instance, a recent study addressed patients with allergic rhinitis and adenoid hypertrophy^[23]^) and the relationship between single-allergen immunotherapy and sensitization to other allergens.^[34]^ Further, more detailed investigations into the mechanisms underlying the effect of SLIT administration are imperative.

## Acknowledgments

Editorial support, in the form of medical writing, assembling tables, and creating high-resolution images based on authors’ detailed directions, collating author comments, copyediting, fact checking, and referencing, was provided by Editage, Cactus Communications.

## Author contributions

**Conceptualization:** Teruyuki Kajiume.

**Data curation:** Teruyuki Kajiume.

**Formal analysis:** Teruyuki Kajiume.

**Investigation:** Teruyuki Kajiume.

**Methodology:** Teruyuki Kajiume.

**Project administration:** Teruyuki Kajiume.

**Resources:** Teruyuki Kajiume.

**Software:** Teruyuki Kajiume.

**Validation:** Teruyuki Kajiume.

**Visualization:** Teruyuki Kajiume.

**Writing – original draft:** Teruyuki Kajiume.

**Writing – review & editing:** Teruyuki Kajiume.
